# Spatial and Temporal Heterogeneity of Tumor-Infiltrating Lymphocytes in Advanced Urothelial Cancer

**DOI:** 10.3389/fimmu.2021.802877

**Published:** 2022-01-03

**Authors:** Sandra van Wilpe, Mark A. J. Gorris, Lieke L. van der Woude, Shabaz Sultan, Rutger H. T. Koornstra, Antoine G. van der Heijden, Winald R. Gerritsen, Michiel Simons, I. Jolanda M. de Vries, Niven Mehra

**Affiliations:** ^1^ Department of Medical Oncology, Radboud University Medical Center, Nijmegen, Netherlands; ^2^ Department of Tumor Immunology, Radboud Institute for Molecular Life Sciences, Radboud University Medical Center, Nijmegen, Netherlands; ^3^ Oncode Institute, Nijmegen, Netherlands; ^4^ Department of Medical Oncology, Rijnstate Hospital, Arnhem, Netherlands; ^5^ Department of Urology, Radboud Institute for Molecular Life Sciences, Radboud University Medical Center, Nijmegen, Netherlands; ^6^ Department of Pathology, Radboud Institute for Molecular Life Sciences, Radboud University Medical Center, Nijmegen, Netherlands

**Keywords:** urothelial cancer, tumor-infiltrating lymphocytes, spatial heterogeneity, longitudinal changes, biomarkers

## Abstract

Checkpoint inhibitors targeting PD-(L)1 induce objective responses in 20% of patients with metastatic urothelial cancer (UC). CD8^+^ T cell infiltration has been proposed as a putative biomarker for response to checkpoint inhibitors. Nevertheless, data on spatial and temporal heterogeneity of tumor-infiltrating lymphocytes in advanced UC are lacking. The major aims of this study were to explore spatial heterogeneity for lymphocyte infiltration and to investigate how the immune landscape changes during the disease course. We performed multiplex immunohistochemistry to assess the density of intratumoral and stromal CD3^+^, CD8^+^, FoxP3^+^ and CD20^+^ immune cells in longitudinally collected samples of 49 UC patients. Within these samples, spatial heterogeneity for lymphocyte infiltration was observed. Regions the size of a 0.6 tissue microarray core (0.28 mm^2^) provided a representative sample in 60.6 to 71.6% of cases, depending on the cell type of interest. Regions of 3.30 mm^2^, the median tumor surface area in our biopsies, were representative in 58.8 to 73.8% of cases. Immune cell densities did not significantly differ between untreated primary tumors and metachronous distant metastases. Interestingly, CD3^+^, CD8^+^ and FoxP3^+^ T cell densities decreased during chemotherapy in two small cohorts of patients treated with neoadjuvant or palliative platinum-based chemotherapy. In conclusion, spatial heterogeneity in advanced UC challenges the use of immune cell infiltration in biopsies as biomarker for response prediction. Our data also suggests a decrease in tumor-infiltrating T cells during platinum-based chemotherapy.

## Introduction

Whereas cisplatin-based chemotherapy remains the standard first-line treatment for patients with metastatic urothelial cancer (UC), immune checkpoint inhibitors have become available as an additional treatment option for these patients in the last few years. Checkpoint inhibitors targeting programmed cell death protein-1 (PD-1) or its ligand (PD-L1) are currently available for those that progressed on first-line platinum-based chemotherapy and those that are cisplatin-ineligible and have a PD-L1 positive tumor. Although anti-PD-(L)1 is able to induce durable responses in some patients, objective responses and disease control are achieved in only 21.1% and 38.5% of patients with metastatic UC, respectively (1).

Researchers are now seeking to improve the efficacy of checkpoint inhibitors by combining it with chemotherapy or using it in earlier disease stages. Recently, a randomized, phase III trial demonstrated that maintenance therapy with PD-L1 inhibitor avelumab significantly prolonged overall survival (OS) compared to watchful waiting in patients who achieved a response or stable disease with first-line chemotherapy (21.4 vs 14.3 months) (2). Moreover, a phase III trials in patients with localized muscle-invasive UC has shown that nivolumab improves disease-free survival in the adjuvant setting (20.8 versus 10.8 months) (3). In light of these developments, there is a need for a thorough understanding on how the responsiveness to checkpoint inhibitors in UC changes during the disease course and whether or not the immune infiltrate can be used to predict response to checkpoint inhibitors.

In UC, CD8^+^ T cell infiltration has been associated with response to checkpoint inhibitors (4–7). Among 212 metastatic UC patients, objective responses to PD-1 inhibitor nivolumab were observed in 25.5% and 11.3% of patients with high and low CD8^+^ T cell infiltration, respectively (4). Results from the ABACUS trial, a phase II trial investigating the efficacy of neoadjuvant atezolizumab, indicate that pretreatment CD8^+^ T cell infiltration is also associated with higher pathologic complete response rates to neoadjuvant atezolizumab [40% vs 20% (high vs low)] (6). Nevertheless, the spatial heterogeneity of T cell infiltration has not been studied in muscle-invasive or metastatic UC. A heterogenous distribution of CD8^+^ T cells within a tumor mass may impede accurate evaluation of immune cell infiltration, especially when using small biopsies (8). In addition, there may be substantial variation in immune density depending on the site of metastases, which might limit the predictive value of CD8^+^ T cell infiltration when a single cutoff value is used (9).

Considering the association between CD8^+^ T cell infiltration and response to checkpoint inhibitors and the interest in the use of checkpoint inhibitors in other disease settings, knowledge on the evolvement of the immune infiltrate during the disease course is desired. Several studies have investigated the relationship between T cell infiltration and disease stage in localized UC. These studies did not find a strong association with tumor stage and/or grade (10). Little is known, however, on the density of the immune infiltrate in metastatic UC compared to localized UC or on the changes in individual patients during the disease course. Apart from the changes during progression from localized to metastatic disease, it is also important to understand how platinum-based chemotherapy affects the immune infiltrate.

In this exploratory study, we evaluated the density of total (CD3^+^), cytolytic (CD8^+^) and regulatory (FoxP3^+^) T cells and B cells (CD20^+^) in longitudinally collected samples of 49 UC patients using multiplex immunohistochemistry (mIHC). The major aims of this study were to explore spatial heterogeneity for lymphocyte infiltration, both between different tissue sites and within individual tumor masses, and to investigate how the immune landscape changes during the disease course.

## Material and Methods

### Patients and Samples

In this study, we used archival tumor samples of 49 patients treated for metastatic UC at the Radboud University Medical Center between 2016 and 2019. The study population comprised both patients with upper and lower urinary tract disease.

Because one of our primary aims was to study longitudinal changes, multiple samples per patient were included, if applicable. For the assessment of lymphocyte infiltration in the primary tumor, we preferably used tissue resected during cystectomy and/or (nefro)ureterectomy. When this was unavailable, we used tissue obtained during transurethral resection (TURT) instead. Whenever a patient had received neoadjuvant chemotherapy (NAC) before radical cystectomy, we included both tissue obtained before (TURT) and after NAC (cystectomy). In the rare cases that a patient developed two primary tumors (in different parts of the urinary tract), tissue of both primary tumors was used. Further, if the patient had local lymph node metastases at the time of surgery, we also included tissue of a synchronous lymph node metastasis. To assess lymphocyte infiltration in distant metastases, archival tumor tissue obtained in the metastatic setting was used. If a patient had undergone multiple biopsies in the metastatic setting, all samples were used for mIHC.

This study was approved by the local Radboudumc medical ethical committee (file number 2017-3934). All patients provided written informed consent to scientific use of leftover tissue, unless deceased.

### Immunohistochemistry

Formalin-fixed paraffin-embedded tissue blocks were cut into 4 µm thick tissue sections. Consecutive tissue sections were used for hematoxylin and eosin (H&E), 7-color mIHC, GATA3 and cytokeratin 5/6 (KRT5/6) stainings.

The 7-color mIHC stainings were performed using primary antibodies against CD3 (RM-9107, Thermo Fisher, clone Sp7), CD8 (M7103, DAKO, clone C8/144B), FoxP3 (14-4777, eBioscience, clone 236A/E7), CD20 (MS-340-S, Thermo Fisher, clone L26), CD45RO (MS-112, Thermo Fisher, clone UCHL-1) and pan cytokeratin (ab86734, abcam, clone AE1/AE3 +5D3). In this panel, pan cytokeratin served as tumor marker. For details on antibody order and fluorochrome pairing, we refer to **Supplementary Table 1**. Methods for panel optimization and validation have previously been described (11).

The fully automated BOND-RX IHC stainer (Leica Biosystems) was used to perform the 7-color mIHC stainings. First, slides were deparaffinized using Bond Dewax Solution (AR9222, Leica). Before application of the primary antibody, antigen retrieval was performed in Bond Epitope Retrieval 2 solution (AR9640, Leica) at 95°C for 20 minutes. After this, slides were incubated in Opal antibody diluent (ARD1001EA, PerkinElmer) for 10 minutes to reduce nonspecific background staining. Next, the primary antibody was applied for 1 hour at room temperature. Slides were then washed three times with Bond Wash Solution (AR9590, Leica), before applying Opal Polymer anti-mouse/anti-rabbit HRP secondary antibodies (NEL801001KT, PerkinElmer). After this, slides were washed again three times, Opal TSA substrate was applied in a 1:50 dilution (Opal520, 540, 570, 620 and 690) or a 1:200 dilution (Opal650) for 10 minutes and the slides were washed again (NEL801001KT, PerkinElmer). This process was repeated for all six markers. Finally, DAPI nuclear counterstain, diluted in TBST, was applied manually for 5 minutes (NEL801001KT, PerkinElmer) and slides were embedded in Fluormount-G (0100-01, ITK).

DAB stainings for GATA3 (390M-16, Cell Marque, clone L50-823, 1:100) and KRT5/6 (m7237, DAKO Agilent, clone D5/16 B4, 1:160) were performed using the semi-automated Lab Vision Autostainer (Thermo Scientific). Antigen retrieval was performed with Target Retrieval Solution, High pH (K8004, DAKO) at 97°C for 10 minutes. After application of the primary antibody, visualization was achieved with EnVision Flex, High pH (K8000, DAKO).

### Tissue Imaging and Analysis

The H&E, GATA3 and KRT5/6 stainings were visually assessed by a dedicated genitourinary pathologist. H&E staining served to confirm the presence of tumor tissue and to evaluate the histological subtype. In the case of discrepancies with the original pathology report regarding histological subtype, all available tissue sections of the particular specimen were reevaluated. The GATA3 and KRT5/6 stainings were performed in order to classify tumors into basal and luminal subtypes (12–14). Although molecular subtyping is mostly performed using RNA sequencing data, literature supports that GATA3 and KRT5/6 can be used to reliably distinguished luminal and basal subtypes (13, 14). The GATA3 and KRT5/6 stainings were evaluated for both the intensity of staining (score 0-3) and the percentage (0-100) of positive tumor cells, as previously described (15). An H-score was calculated by multiplying the percentage of positive cells by the intensity (range 0-300). Tumors were considered GATA3 and/or KRT5/6 positive if the H-score was ≥20 (16, 17).

**Figure 1:**
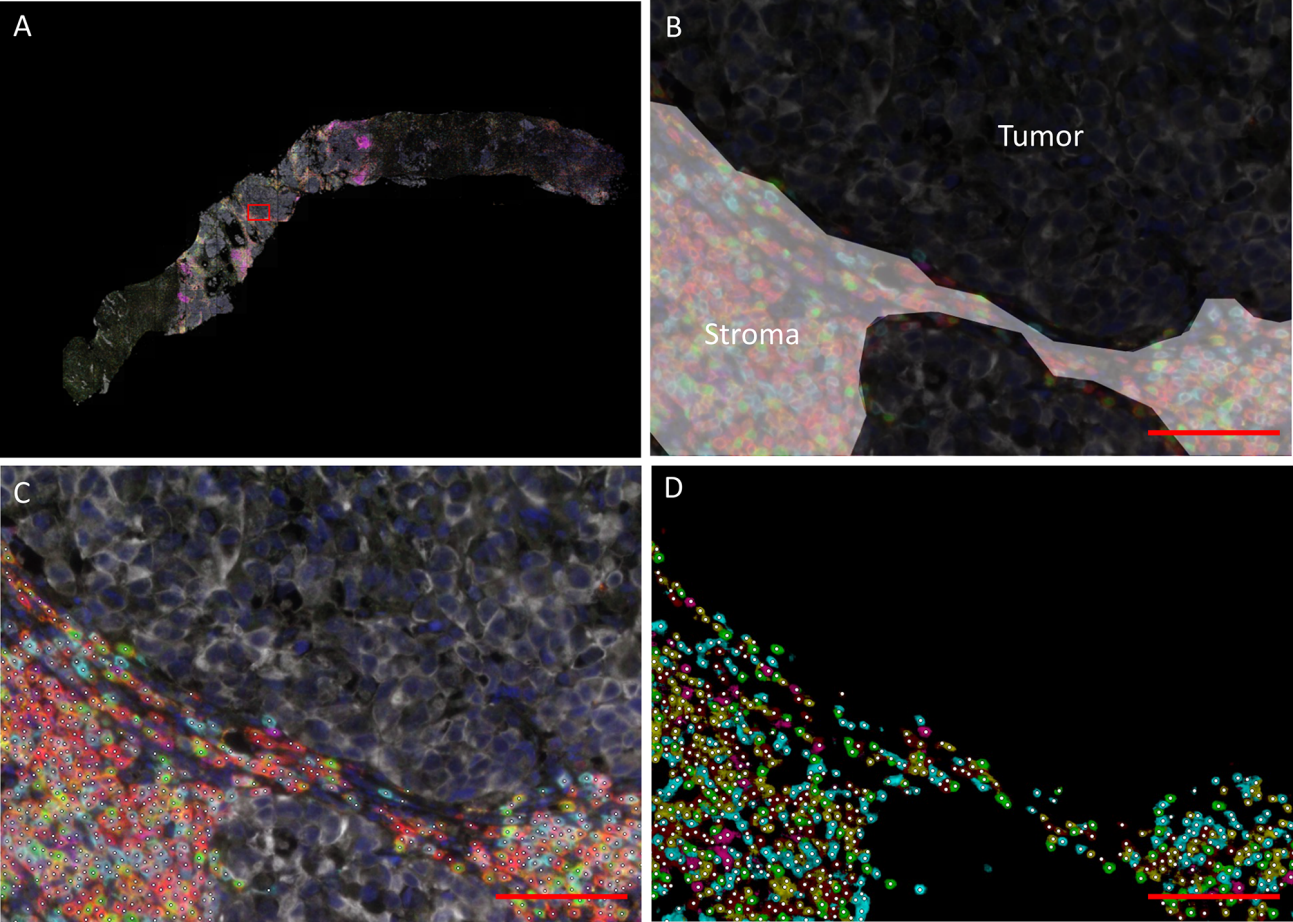
Data processing and analysis of mIHC images. **(A)** Overview of a biopsy after spectral unmixing by PerKinElmer inForm^®^ image-analysis. The rectangle indicates the region depicted in **(B–D) (B)** Tissue segmentation by PerkinElmer inForm. An algorithm was trained based on the expression of pan cytokeratin, DAPI and autofluorescence to discriminate between tumor (black) and stroma (white). C/D. Cell segmentation **(C)** and phenotyping **(D)**. A neural network was trained to identify T cells and B cells (white dots) based on the expression of the seven immunohistochemistry markers (red = CD3; cyan = CD8; green = FoxP3; magenta = CD20; yellow = CD45RO; white = tumor marker; dark blue = DAPI). The scalebar represents a distance of 100 µm.

Lymphocyte infiltration was quantified using a fully automated approach. The mIHC-stained slides were scanned using the PerkinElmer Vectra^®^ 3 Automated Quantitative Pathology Imaging System, with software version 3.0.4. Single stainings were used to set the exposure times. Slides were first scanned at 4x magnification. Using the PerkinElmer Phenochart software (version 1.0.9), tumor regions plus one surrounding region of stroma (669 × 500 µm) were selected for imaging at 20X magnification (**Figure 1A**, **Supplementary Figure 1**). PerkinElmer inForm^®^ image-analysis (software version 2.4.2) was used for spectral unmixing, removal of autofluorescence signal and tissue segmentation. For tissue segmentation, an algorithm was trained based on the expression of pan cytokeratin, DAPI and autofluorescence to discriminate between tumor, stroma and background (**Figure 1B**). Images and tissue phenotyping data were then exported from inForm for cell segmentation and phenotyping by an in-house developed neural network (**Supplementary Figure 2**) (18). The neural network was trained for this specific mIHC panel by annotating over 40.000 cells across samples of different cancer types, including urothelial cancer. Shortly, the neural network identifies T cells and B cells based on the expression of the seven IHC markers and predicts for each identified cell which of the markers is expressed (**Figures 1C, D**). The data generated by the neural network were exported in Flow Cytometry Standard (FCS) files. Subsequently, cell populations were gated in FlowJo (version 10, Tree Star Inc., Ashland, OR, USA) using the predicted marker expression of the neural network (**Supplementary Figure 3**).

### Immune Cell Subsets

In this study, we differentiated between intratumoral immune cells and immune cells located in the surrounding stroma. If the tumor or stroma region was smaller than 0.10 mm^2^, the sample was excluded from analyses for that specific region. In this paper, we present data on 4 immune cell subsets: total T cells (CD3^+^), cytotoxic T cells (CD8^+^), regulatory T cells (FoxP3^+^) and B cells (CD20^+^). Unfortunately, we were not able to accurately distinguish CD45RO^+^ from CD45RO^-^ cells. Therefore, CD45RO^+^ was excluded from analysis.

### Selection of Tumor Regions for the Analysis of Heterogeneity

Previous studies evaluating immune cell infiltration in UC have frequently used tissue microarrays (TMA) (12, 19–22) or selected only a limited number of fields (mostly 0.07 mm^2^/field) from stained whole-slides for assessment (23–26). To study whether these small tissue sections can provide a representative sample, we randomly selected four tumor regions of 0.28 mm^2^ (similar to a 0.6 mm diameter core on a TMA). These regions were predominantly segmented as tumor tissue by inForm tissue segmentation, but small stromal bands (small areas in between tumor cells without pan cytokeratin expression; **Supplementary Figure 4**) were allowed. If a sample only contained small islets of tumor cells or was too small to select four non-overlapping tumor regions, the sample was excluded from analysis of heterogeneity.

In addition, to evaluate whether biopsies are representative for immune cell infiltration in a tumor, we randomly selected four tumor regions the size of the median tumor surface area in our biopsies (3.30 mm^2^). As many samples were too small to select four of these larger tumor regions, these regions could only be selected in a minority of samples (n=20).

### Statistical Analysis

First, we evaluated whether lymphocyte infiltration differed depending on the tissue of origin. To assess this, we selected all tissue sites for which we had at least 5 samples available. For each cell subset (CD3^+^, CD8^+^, FoxP3^+^, CD20^+^), the intratumoral and stromal cell densities per tissue site were visualized in boxplots. A Kruskal-Wallis test was performed to test whether there were significant differences (p-value ≤ 0.05, no correction for multiple testing) between tissue sites. If significant, a *post-hoc* Dunn’s test with Bonferroni correction was performed for pairwise comparisons.

Next, we assessed heterogeneity within samples. For this, four tumor regions of 0.28 mm^2^ were selected per sample, as described above. To gain insight into the relevance of the observed heterogeneity, we classified the samples into four quartiles based on the mean cell densities of the four regions and determined the percentage of regions that was classified into the same (correct) quartile. This procedure was repeated for the larger tumor regions (3.30 mm2).

Data from paired tumor samples were used to study longitudinal changes in lymphocyte density within individual patients. First, immune cell infiltration in untreated primary tumors was compared with immune cell infiltration in metachronous metastases. If there had been two primary tumors or if multiple biopsies from the metastatic setting were available, the cell densities of these samples were averaged. Differences were analyzed using a Wilcoxon signed-rank test. Again, a p-value below 0.05 was considered statistically significant. Subsequently, samples obtained before and after chemotherapy were compared in two cohorts of patients (neoadjuvant and palliative chemotherapy). Because of the small sample size, no statical tests were performed. The median change and range were used to describe changes during chemotherapy.

All statistical analyses were performed in R version 4.0.2.

## Results

### Patient Characteristics and Samples

Immune cell infiltration was assessed in 111 longitudinally collected samples of 49 patients with metastatic UC. The study population consisted of both patients with upper (n=11) and lower urinary tract disease (n=35). In three cases the origin of the metastases was unclear, because the patient presented with metastatic disease without a detectable primary tumor (n=1) or because the patient had a history of both invasive UC of the bladder and upper urinary tract (n=2). All patients had a tumor of urothelial origin. Sixteen patients (32.7%) had a component of divergent differentiation. This mainly concerned squamous (n=8) or sarcomatoid differentiation (n=2). Further patient characteristics are shown in **Table 1**. An overview of all available samples is given in **Supplementary Figure 5**.

**Table 1:** Patient characteristics.

Age in years at diagnosis MI-UC or mUC – median (range)	66 (23 - 77)
Sex – no. (%)	
Male	40 (81.6)
Female	9 (18.4)
Origin of disease – no. (%)	
Bladder	35 (71.4)
Ureter or renal pelvis	11 (22.4)
Both	2 (4.1)
Unknown	1 (2.0)
Stage at first diagnosis – no. (%)	
NMI-UC	18 (36.7)
Localized MI-UC	23 (46.9)
mUC	8 (16.3)
TNM stage at diagnosis MI-UC or mUC – no. (%)	
T2N0M0	6 (12.2)
T3-4N0M0	14 (28.6)
N1-3M0	14 (28.6)
M1	15 (30.6)
Interval between localized MI-UC and mUC in months – median (range)*	14.5 (3 – 81)

*For those that received curative treatment for localized MI-UC before the development of distant metastases (n=34). NMI-UC, non-muscle invasive urothelial cancer; MI-UC, muscle-invasive urothelial cancer; mUC, metastatic urothelial cancer.

### General Overview of Immune Cell Infiltration Across All Samples

Across all 111 samples, the median surface area of the intratumoral and stromal compartment was 7.23 mm^2^ (range 0.14 – 150.40) and 10.1 mm^2^ (range 0.0083 – 117.47), respectively. In one sample, the stromal cell density could not be assessed due to a stromal surface of less than 0.10 mm^2^. The intratumoral and stromal surface area in biopsies was smaller compared to samples that were obtained through surgery (TURT, cystectomy or (nefro)ureterectomy). The median surface area of the intratumoral and stromal regions in biopsies was 3.30 mm^2^ (range 0.14 – 14.93) and 1.51 mm^2^ (range 0.0083 – 20.78), respectively.

For all immune cell subsets, higher cell densities were observed in the stroma than in the tumor compartment. The median densities of CD3^+^, CD8^+^, FoxP3^+^ and CD20^+^ cells in the tumor compartment were 376.6 cells/mm^2^ (range 28.1 – 3497.6), 140.0 cells/mm^2^ (range 1.1 – 2511.9), 71.0 cells/mm^2^ (range 2.5 – 539.8) and 8.3 cells/mm^2^ (range 0 – 1494.8), respectively. Median cell counts in stroma were 1255.4 cells/mm^2^ (99.5 – 8959.5), 394.0 cells/mm^2^ (range 19.3 – 2539.9), 251.7 cells/mm^2^ (range 29.2 – 1760.9) and 124.9 cells/mm^2^ (range 0 – 3784.0). CD20^+^ cells were frequently clustered in tertiary lymphoid structures (TLS), which can be recognized as CD20^+^ B cell follicles, adjacent to a CD3^+^ T cell zone (27).

In total, 105 samples could be assessed for GATA3 and KRT5/6 expression. Of these samples, 25 were positive for GATA3 and KRT5/6 (23.8%), 55 were only positive for GATA3 (luminal; 52.4%), and 15 were only positive for KRT5/6 (basal; 14.3%). Ten samples were negative for both markers (9.5%).

### Differences per Tissue Site

**Figure 2:**
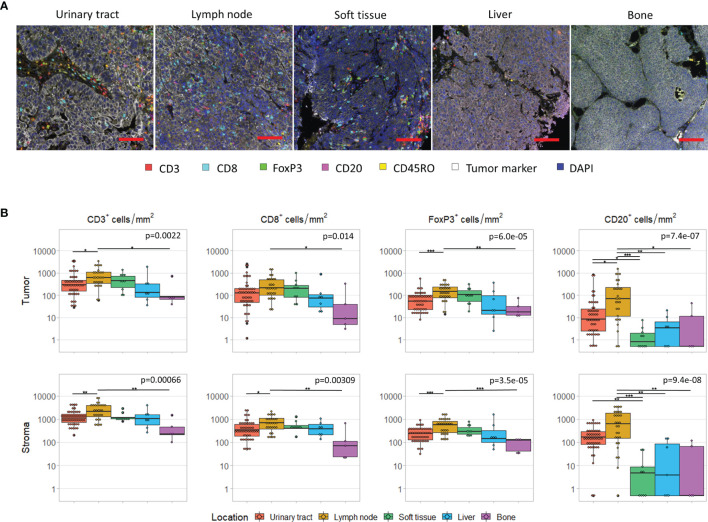
Immune cell infiltrate per tissue site. **(A)** Multiplex immunohistochemistry images of the five most frequent tissue sites. The scalebar represents a distance of 100 µm. **(B)** CD3^+^, CD8^+^, FoxP3^+^ and CD20+ cell density in the tumor (upper panel) and stroma compartment (lower panel) per tissue site. For all cell subsets, we observed significant differences between tissue sites, both in the stromal and tumoral compartments (Kruskal-Wallis test, p < 0.05). Black lines indicate significant differences between pairs (Dunn’s test, *p < 0.05, **p ≤ 0.01, ***p ≤ 0.001). In some patients, no CD20+ cells were present. To enable visualization of cell densities on a log scale, the CD20+ densities of these patients was replaced by 0.5 cells/mm2 (~lowest value in this plot).

First, we assessed the differences in immune cell infiltration per tissue site. For five tissue sites, we had at least five samples available, that is urinary tract (n=50), lymph node (n=29), soft tissue (n=10), liver (n=7) and bone (n=5). The cell densities per tissue site are shown in **Figure 2**. For all cell subsets, we observed significant differences between tissue sites (p < 0.05). Interestingly, this was not only observed in the stroma (**Figure 2B**, lower panel), but also in the tumoral compartment (**Figure 2B**, upper panel). Pairwise comparisons revealed that the densities of intratumoral CD3^+^ and FoxP3^+^ T cells were significantly higher in lymph node metastases compared to bone metastases (CD3^+^ cells: p=0.025; FoxP3^+^: p=0.0006) or tumors located in the urinary tract (CD3^+^ cells: p=0.022; FoxP3^+^: p=0.0041). A significant difference between lymph node and bone metastases was also observed for intratumoral CD8^+^ T cells (p=0.035). Finally, the intratumoral CD20^+^ cell density significantly differed between lymph node metastases and lesions in all other locations (urinary tract: p=0.014; bone: p=0.028; liver: p=0.0063; soft tissue: p<0.0001) as well as between urinary tract and soft tissue lesions (p=0.015). Nevertheless, the median cell densities in the latter two locations were both very low (8.4 versus 0.7 cells/mm^2^). Comparable results were obtained for stromal immune cell densities (**Figure 2B**).

GATA3 and KRT5/6 expression also differed between the five tissue sites. Whereas 51-52% of urinary tract and lymph node were classified as luminal, this was the case for 30% of soft tissue, 71.4% of liver and 100% of bone lesions. Nevertheless, when we selected only the luminal samples, we still observed large differences between tissue sites, with lymph node metastases still containing the most dense and bone metastases the least dense infiltrates (data not shown). The basal subgroup was too small to compare tissue sites.

TLSs, which can only be assessed in non-lymphoid tissues, were observed in most urinary tract samples (43/50), but were infrequent in samples from other tissue sites (liver: 1/7; soft tissue: 0/10; bone: 0/5). This difference may be explained by the larger area of stroma in urinary tract samples, as TLSs were mostly located in the stromal compartment.

**Figure 3:**
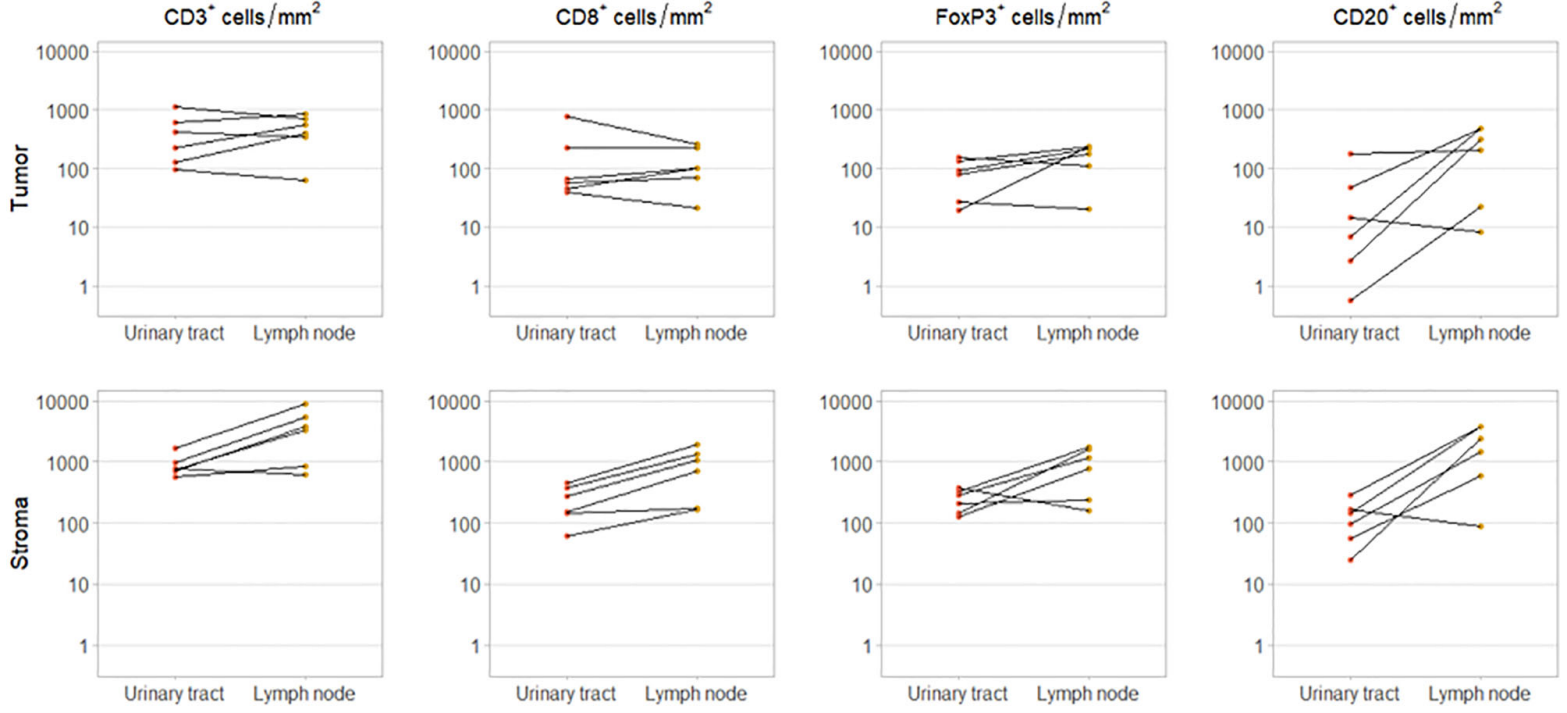
Immune cell infiltration in primary tumor and synchronous lymph node metastasis. Intratumoral (upper panel) and stromal (lower panel) CD3^+^, CD8^+^, FoxP3^+^ and CD20^+^ cell densities in paired urinary tract and lymph node samples. These samples were obtained during the same procedure.

For six patients, we had obtained tissue from both the primary tumor and a synchronous local lymph node metastases, resected during the same procedure. In these patients, intratumoral CD3^+^ and CD8^+^ cell densities did not clearly differ between urinary tract and lymph node samples. However, intratumoral FoxP3^+^ and CD20^+^ cell densities were higher in the lymph node metastases in four and five out of six patients, respectively. As expected stromal immune cell densities were also higher in the lymph nodes (**Figure 3**).

### Heterogeneity Within Samples

**Figure 4:**
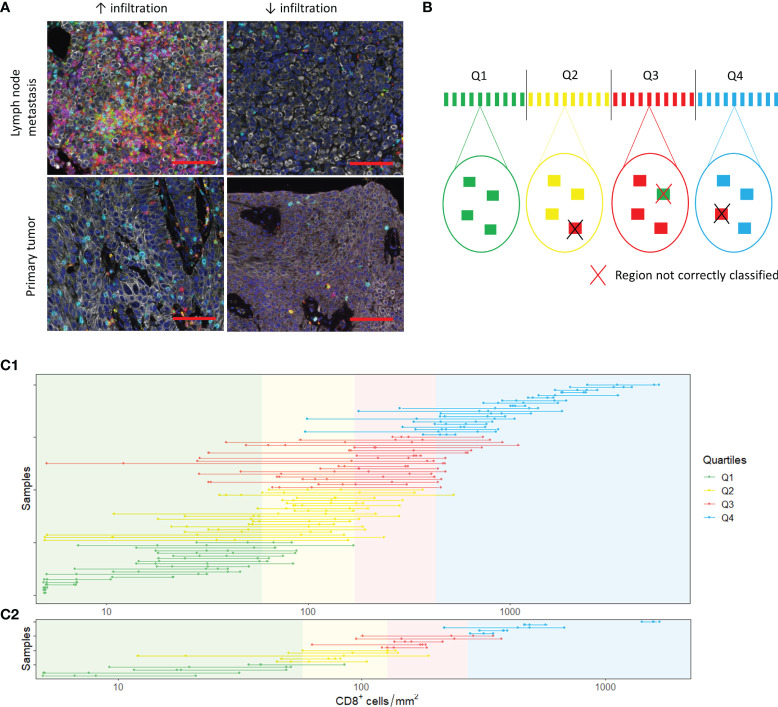
Spatial heterogeneity. **(A)** Spatial heterogeneity in a lymph node metastasis (upper panel) and a primary tumor (lower panel). Images on the right and left side are derived from the same sample. The scalebar represents a distance of 100 µm. **(B)** Analysis of heterogeneity. In each sample, four tumor regions were selected. The mean cell density of these four regions was calculated. Based on the mean cell densities of the samples, quartiles were defined (upper part of the figure). Next, we evaluated whether the small regions were representative by checking whether the individual regions belonged to the same quartiles as the sample mean. **(C)** CD8^+^ T cell densities in the selected 0.28 mm2 (C1) and 3.30 mm^2^ (C2) regions. The dots show the cell densities of the regions. The four regions of one sample are interconnected by a line. In some regions, no CD8^+^ cells were present. To enable visualization of cell densities on a log scale, the CD8^+^ densities of these patients was replaced by 5 cells/mm2 (~lowest value in C1 and C2).

Next, we assessed heterogeneity within tumor samples. On visual inspection, we observed substantial heterogeneity within samples (**Figure 4A**). To study whether small TMA cores can provide a representative sample, we randomly selected four tumor regions of 0.28 mm^2^ (**Figure 4B**). We were able to select four regions in 80 out of 111 samples. Of these samples, 45 were derived from the urinary tract and 35 from metastatic sites. The cell densities per region are displayed in **Figure 4C1** (CD8^+^ cells) and **Supplementary Figures 5A, 6A** and **7A** (CD3^+^, FoxP3^+^ and CD20^+^ cells). The median difference in cell count/mm^2^ between the region with the highest and the region with the lowest cell density was 515.40 for CD3^+^ cells (range 10.75 - 5884.79), 221.57 for CD8^+^ cells (range 0 – 2957.72), 136.16 for FoxP3^+^ cells (range 0 – 155.88) and 16.07 for CD20^+^ cells (range 0 – 2264.49).

For each immune cell subset, we categorized the 80 samples into quartiles based on the mean cell densities of the four randomly selected regions. Subsequently, we determined whether the four regions were classified into the same quartile. Regions were accurately classified in 64.4%, 63.4%, 60.6% and 71.6% of cases for CD3^+^, CD8^+^, FoxP3^+^ and CD20^+^ cells, respectively (**Supplementary Tables 2** and **3**).

To evaluate whether biopsies are representative for immune cell infiltration in a tumor, we repeated the same analysis for tumor regions of 3.30 mm^2^, the median tumor surface area of all our biopsies (**Figure 4C2**, **Supplementary Figures 5B, 6B** and **7C**). We were able to select four regions of 3.30 mm^2^ in 20 samples. Most of these samples were derived from the urinary tract (n=14). The median difference in cell count/mm^2^ between the region with the highest and the region with the lowest cell density was low compared to the 0.28 mm^2^ regions: 188.97 for CD3^+^ cells (range 38.46 – 659.73), 79.76 for CD8^+^ cells (range 17.87 – 460.34), 48.49 for FoxP3^+^ cells (range 8.48 – 343.99) and 5.45 for CD20^+^ cells (range 0 – 160.31). Nevertheless, the correct classification rate for CD3^+^ and CD8^+^ cells was only slightly higher (CD3: 71.3%; CD8: 73.8%) and the correct classification rate for FoxP3^+^ and CD20^+^ cells was poorer (FoxP3: 58.8%; CD20: 62.5%) (**Supplementary Tables 4** and **5**).

### Longitudinal Changes—Primary Tumor versus Metastasis

Another aim of this study was to investigate longitudinal changes in tumor-infiltrating lymphocytes within individual patients. First, immune cell infiltration in untreated primary tumors was compared to immune cell infiltration in metachronous metastases. For 28 patients, tissue of the primary tumor and one or more metastases was available. Whereas the cell densities in distant lymph node metastases seemed to be slightly higher compared to primary tumors, overall, there was no significant difference between primary tumor and metastasis for any of the cell subsets. We also did not observe a clear change in the ratio of intratumoral to stromal CD8^+^ cells or in the ratio of FoxP3^+^ to CD8^+^ T cells (**Supplementary Figure 9**).

### Longitudinal Change—Changes During Platinum-Based Chemotherapy

**Figure 5:**
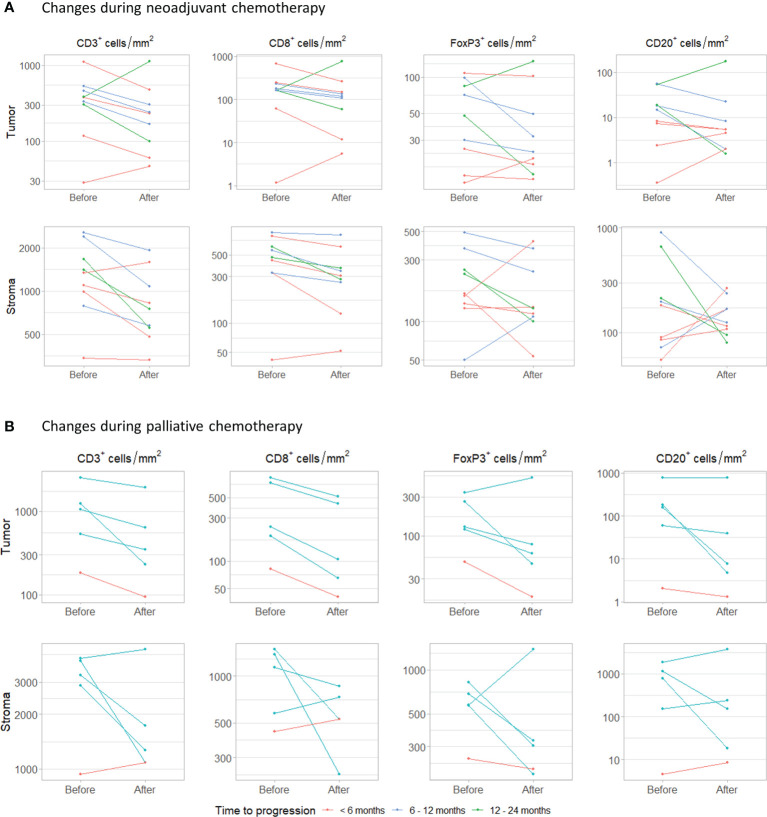
Changes during chemotherapy. **(A)** Changes in intratumoral and stromal CD3^+^, CD8^+^, FoxP3^+^ and CD20^+^ cell density during neoadjuvant chemotherapy. Samples were obtained by transurethral resection (before) and cystectomy (after). **(B)** Changes during palliative platinum-based chemotherapy. Paired samples were obtained from the same tissue site.

Finally, we assessed how the immune infiltrate changed during platinum-based chemotherapy. For nine patients treated with NAC, we had both tumor tissue available obtained before (TURT) and after NAC (cystectomy) (**Figure 5A**). Patients were treated with gemcitabine plus cisplatin (n=4), gemcitabine plus carboplatin (n=4) or dose dense MVAC (n=1) and still had muscle-invasive disease at the time of cystectomy (≥ ypT2a). Four patients progressed <6 months after cystectomy, three within 6-12 months and two within 12- 24 months. Interestingly, intratumoral CD3^+^ and CD8^+^ cell density decreased during NAC in seven out of nine patients. Median changes in CD3^+^ and CD8^+^ cells were -164.78 cells/mm^2^ (range -629.63 to 747.83) and -59.58 cells/mm^2^ (-423.09 to 616.90), respectively. FoxP3^+^ cell density also decreased in seven patients, but these changes were small (median change -6.19 cells/mm2, range -66.93 to 51.99). No clear trend was observed for the CD20^+^ cell density (median change -2.79 cells/mm2, range -34.40 to 124.57). The changes observed in stromal immune cell density were comparable to the changes in the tumor compartment. There was no evident change in the FoxP3^+^/CD8^+^ T cell ratio (**Supplementary Figure 10A**).

In five patients that had received palliative platinum-based chemotherapy, tumor tissue had been obtained from the same metastatic site before and after chemotherapy (**Figure 5B**). Patients were treated with carboplatin plus gemcitabine (n=4) or cisplatin plus gemcitabine (n=1). One patient progressed within 6 months and four within 6-12 months. The interval between the last cycle of chemotherapy and the second biopsy ranged from 2 to 6 months. In all patients, intratumoral CD3^+^ and CD8^+^ cell density decreased. Median change in CD3^+^ and CD8^+^ cell density was -415.58 cells/mm^2^ (range -1003.08 to -89.07) and -134.74 cells/mm^2^ (range -319.55 to -41.45), respectively. Intratumoral FoxP3^+^ and CD20^+^ cell density decreased in four out of five patients. Median changes in FoxP3^+^ and CD20^+^ cell density were -51.71 cell/mm^2^ (range -216.24 to 182.12) and -21.30 cells/mm^2^ (-177.18 to 11.75). Stromal CD3^+^, CD8^+^, FoxP3^+^ and CD20^+^ cell densities decreased in three, three, four and two out of five patients, respectively. Again, no clear trend in the ratio between FoxP3^+^ and CD8^+^ T cells was observed (**Supplementary Figure 10B**).

## Discussion

In this multiplex IHC study, we explored spatial heterogeneity of tumor-infiltrating lymphocytes and investigated how the immune landscape changes during the disease course, using longitudinally collected tumor samples of 49 UC patients. We observed significant differences in immune cell infiltration depending on the tissue site, with the differences being most pronounced between lymph node and bone metastases. Within samples, we also observed substantial spatial heterogeneity. Tumor regions of 3.30 mm^2^, the median tumor surface area in our biopsies, provided a representative sample in only 58.8 to 73.8% of cases, depending on the marker of interest. In this study, we did not observe a clear increase or decrease in immune cell infiltration between untreated primary tumors and distant metastases. We did, however, observe a decrease in T cell infiltration following chemotherapy in two small cohorts of patient treated with neoadjuvant and palliative platinum-based chemotherapy.

One of our main findings is that immune cell infiltration differs depending on the tissue of origin. Across the entire cohort, intratumoral immune cell densities in lymph node metastases were higher than in bone metastases (CD3^+^, CD8^+^, FoxP3^+^ and CD20^+^ cells) or tumors located in the urinary tract (CD3^+^, FoxP3^+^ and CD20^+^ cells). Although the difference in intratumoral CD3^+^ T cell density between urinary tract and lymph node lesions could not be confirmed in a small cohort of patients with simultaneously resected urinary tract and lymph node samples (n=6), we did observe higher intratumoral FoxP3^+^ and CD20^+^ cell densities in lymph node metastases of these patients. In line with our findings, studies in other cancer types have also reported differences in immune cell infiltration between tissue sites (9).

Previous research in UC suggest that CD8^+^ T cell infiltration is associated with response to checkpoint inhibitors. The observed differences between tissue sites suggest that it is not appropriate to use a single cutoff value for CD8^+^ T cell infiltration in patients with UC, complicating the use of CD8^+^ T cells as biomarker in the metastatic setting. Although our data provide a first insight into differences between tissue sites in UC, paired samples from individual patients are needed to confirm that immune cell infiltration within patients really differs depending on the tissue of origin and is not a consequence of differences between patients.

Another important finding of this study is that there is considerable spatial heterogeneity within tumors. Tumor regions of 0.28 mm^2^, the size of a 0.6 TMA, provided a representative sample in 60.6 to 71.6% of cases, depending on the cell subset of interest; tumor regions of 3.30 mm^2^, the median tumor surface area in our biopsies, were representative in 58.8 to 73.8% of cases. Although spatial heterogeneity has been described to distort immune cell quantification in other tumor types (8), data on heterogeneity in UC is limited to one study in non-muscle invasive bladder cancer (19). That study indicated that five and two 0.6 mm tumor cores are needed to provide correct sampling of Ta and T1 tumors, respectively. The authors randomly selected 10 tumor regions of 0.28 mm^2^ and used a bootstrapping approach to simulate an estimation of CD8^+^ infiltration according to the number of regions (1-10) selected. By statistical comparison of interquartile range (IQR) distributions, the minimum number of regions for an accurate estimate of CD8^+^ cell infiltration was determined. Here, the bootstrapping estimate for 10 regions functioned as reference value. A limitation of this method is that, from a mathematical point of view, the IQR is expected to get smaller when the number of selected regions approaches the reference value. Therefore, we decided to use another method to analyze heterogeneity. We classified the samples into four quartiles based on the mean cell densities of the four regions and determined the percentage of regions that was classified into the same quartile.

Although our analysis contributes to a better understanding regarding the significance of the observed heterogeneity, some remarks should be made with respect to the used method. Firstly, the decision to classify samples into quartiles instead of tertiles or quintiles was somewhat arbitrary. However, no generally accepted cutoff value for CD8^+^ T cell infiltration in UC has been defined so far. Secondly, we classified samples into quartiles based on the mean cell density of the four regions instead of the whole slide cell density. We felt that this was more appropriate because there was large variation in whole slide tumor surface area. Moreover, although our selected tumor regions were largely classified as tumor tissue by inForm segmentation, the selected regions often included small stromal bands (areas without pan cytokeratin expression). While it is commonly accepted to consider these small areas part of the tumor compartment (20, 21, 26), this made it difficult to compare the cell densities in manually selected regions with cell density in whole slides. Thirdly, a limitation of our study is that we were only able to select large (3.30 mm^2^) regions in small subset of samples, most of which were derived from the primary tumor. It is unclear whether heterogeneity in primary muscle-invasive tumors is comparable with heterogeneity in distant metastases.

Based on the observed heterogeneity, we conclude that median-sized biopsies do not provide a representative sample for the evaluation of immune cell infiltration. Although biopsies might reveal an association between immune cell infiltration and response to checkpoint inhibitors on a group level, they are likely not appropriate to predict response or prognosis in individual patients. There is a substantial risk that the sample is not representative and that the patient is thus classified in the wrong risk group.

Another aim of this study was to evaluate longitudinal changes in lymphocyte density within individual patients. In this study, the density of tumor-infiltrating immune cells did not clearly decrease or increase during progression to metastatic disease. Nevertheless, we should acknowledge that our cohort is heterogeneous, not only in terms of biopsy site but also in terms of the treatments given between primary tumor and metastasis sampling.

While there was no evident change in T cell density during progression to metastatic disease, we did observe a decrease in CD3^+^, CD8^+^ and FoxP3^+^ T cells during chemotherapy, both in the neoadjuvant and palliative setting. Preclinical evidence has suggested that cisplatin and gemcitabine increase the infiltration and cytotoxic activity of CD8^+^ T cells (28–30), whereas it depletes myeloid-derived suppressor cells (MDSCs) (31–33), FoxP3^+^ T cells (34, 35) and macrophages (36). Studies in human patients with other cancer types, on the other hand, have shown that CD8^+^ T cell infiltration might decrease during chemotherapy (37, 38).

Recently, a randomized, phase III trial demonstrated that maintenance therapy with PD-L1 inhibitor avelumab significantly prolonged OS compared to watchful waiting following response or stable disease to first-line chemotherapy (21.4 vs 14.3 months) (2). This indicates that checkpoint inhibitors can be effective in UC patients that have recently received platinum-based chemotherapy. Although the observed decrease in CD3^+^ and CD8^+^ T cells seems inconsistent with the outstanding efficacy of maintenance therapy after chemotherapy, a concurrent decrease in immune suppressive cells might explain the efficacy of checkpoint inhibitors in the context of decreased CD3^+^ and CD8^+^ T cell infiltration. In this study, we observed a slight decrease in FoxP3^+^ T cells but not in the ratio between FoxP3^+^ and CD8^+^ T cells. We did not study other immune suppressive cells, such as macrophages and MDSCs. It might be interesting to include these immune suppressive cells in a future validation study.

As both chemotherapy cohorts were enriched for patients with poor clinical outcomes following chemotherapy, it is unclear whether the observed decrease in T cell infiltration is generalizable to all UC patients receiving platinum-based chemotherapy. As we compared pre- and post-NAC samples, our NAC cohort did not include patients with a complete response. Moreover, all patients eventually developed metastatic disease. The five patients with paired samples before and after palliative chemotherapy all developed progression within 12 months.

We should acknowledge that the number of patients with pre- and post-chemotherapy samples in our study was small. Validation of our results in a larger, two-armed, cohort is required to confirm our results and to exclude that the observed decrease in T cells is related to the method of tissue collection (TURT versus cystectomy) or results from disease progression (palliative setting).

Although most patients in our cohort eventually received checkpoint inhibitors, a minority of patients underwent a biopsy right before initiation of treatment. This, in combination with the small sample size and the different biopsy locations, prevented us from studying associations between lymphocyte infiltration and response to checkpoint inhibitors.

In conclusion, this exploratory study provides a first insight into spatial and temporal heterogeneity in advanced UC. Our data demonstrate that spatial heterogeneity in UC samples distorts immune cell quantification in median-sized biopsies, challenging the use of immune cell infiltration as prognostic or predictive biomarker. In addition, our results indicate that CD3^+^, CD8^+^ and FoxP3^+^ cell densities decrease during treatment with platinum-based chemotherapy.

## Data Availability Statement

The raw data supporting the conclusions of this article will be made available by the authors, without undue reservation.

## Ethics Statement

The studies involving human participants were reviewed and approved by Radboudumc Medical Ethical Committee. All patients provided written informed consent to the scientific use of leftover tissue, unless deceased.

## Author Contributions

SW and NM participated in the conceptualization. SW, MG, LW, SS, and IV participated in the methodology. SW participated in the formal analysis. SW and MS participated in the investigation. SW, MG, LW, SS, IV, and NM provided resources. SW participated in the data curation. SW participated in the writing—original draft preparation. All authors participated in the writing—review and editing. SW participated in the visualization. NM participated in the supervision. All authors contributed to the article and approved the submitted version.

## Conflict of Interest

The authors declare that the research was conducted in the absence of any commercial or financial relationships that could be construed as a potential conflict of interest.

## Publisher’s Note

All claims expressed in this article are solely those of the authors and do not necessarily represent those of their affiliated organizations, or those of the publisher, the editors and the reviewers. Any product that may be evaluated in this article, or claim that may be made by its manufacturer, is not guaranteed or endorsed by the publisher.
