# Supplementary material for: Spatial and Temporal Heterogeneity of Tumor-Infiltrating Lymphocytes in Advanced Urothelial Cancer
Source: Front Immunol. 2022 Jan 3;12:802877. doi: 10.3389/fimmu.2021.802877 (PMC8761759; doi:10.3389/fimmu.2021.802877)
Supplement: Supplementary file 1 [file DataSheet_1.docx]

Supplementary Material

# Supplementary Figures and Tables

## Supplementary Tables

**Supplementary table 1.** 7-color mIHC panel

| **Order** | **Marker** | **Clone** | **Dilution** | **Opal** | **Dilution** |
| --- | --- | --- | --- | --- | --- |
| 1 | CD45RO | UCHL-1 | 1:3000 | 620 | 1:50 |
| 2 | CD8 | C8/144B | 1:200 | 690 | 1:50 |
| 3 | CD20 | L26 | 1:600 | 570 | 1:50 |
| 4 | CD3 | Sp7 | 1:200 | 520 | 1:50 |
| 5 | FoxP3 | 236A/E7 | 1:100 | 540 | 1:50 |
| 6 | Pan cytokeratin | AE1/AE3 +5D3 | 1:1500 | 650 | 1:200 |

**Supplementary table 2.** Quartiles 0.28 mm^2^ regions

|  | **Q1** | **Q2** | **Q3** | **Q4** |
| --- | --- | --- | --- | --- |
| CD3 | 0 – 176.19 | 176.19 – 481.94 | 481.94 – 911.53 | 911.53 - 6044.35 |
| CD8 | 0 – 59.06 | 59.06 – 169.94 | 169.94 – 431.63 | 431.63 - 4129.21 |
| FoxP3 | 0 – 41.48 | 41.48 – 105.88 | 105.88 – 193.59 | 193.59 - 1031.57 |
| CD20 | 0 | 0 – 7.14 | 7.14 – 44.71 | 44.71 - 1282.68 |

**Supplementary table 3.** Classification 0.28 mm^2^ regions

|  | **Q1** | | **Q2** | | **Q3** | | **Q4** | | **Correct classification** |
| --- | --- | --- | --- | --- | --- | --- | --- | --- | --- |
|  | **Correct** | **Total** | **Correct** | **Total** | **Correct** | **Total** | **Correct** | **Total** |  |
| CD3 | 71 | 106 | 41 | 74 | 26 | 52 | 68 | 88 | 64.4 % |
| CD8 | 69 | 110 | 34 | 70 | 29 | 53 | 71 | 87 | 63.4 % |
| FoxP3 | 65 | 105 | 37 | 77 | 27 | 47 | 65 | 91 | 60.6 % |
| CD20 |  |  | 147* | 194* | 33 | 62 | 49 | 64 | 71.6 % |

* More than a quarter of the regions had CD20^+^ cell counts of 0; Therefore, Q1 and Q2 are combined.

**Supplementary table 4.** Quartiles 3.30 mm^2^ region

|  | **Q1** | **Q2** | **Q3** | **Q4** |
| --- | --- | --- | --- | --- |
| CD3 | 0 – 146.12 | 146.12 – 303.83 | 303.83 – 521.39 | 521.39 - 2497.22 |
| CD8 | 0 – 57.31 | 57.31 – 127.28 | 127.28 – 273.02 | 273.02 - 1544.08 |
| FoxP3 | 0 – 30.65 | 30.65 – 75.54 | 75.54 – 112.16 | 112.16 - 422.83 |
| CD20 | 0 – 1.06 | 1.06 – 2.95 | 2.95 – 9.05 | 9.05 – 307.34 |

**Supplementary table 5.** Classification 3.30 mm^2^ regions

|  | **Q1** | | **Q2** | | **Q3** | | **Q4** | | **Correct classification** |
| --- | --- | --- | --- | --- | --- | --- | --- | --- | --- |
|  | **Correct** | **Total** | **Correct** | **Total** | **Correct** | **Total** | **Correct** | **Total** |  |
| CD3 | 18 | 24 | 10 | 15 | 11 | 16 | 18 | 25 | 71.3 % |
| CD8 | 19 | 26 | 8 | 14 | 12 | 18 | 19 | 22 | 73.8 % |
| FoxP3 | 17 | 24 | 10 | 18 | 6 | 15 | 14 | 23 | 58.8 % |
| CD20 | 20 | 33 | 10 | 15 | 6 | 15 | 14 | 17 | 62.5 % |

## Supplementary Figures

**
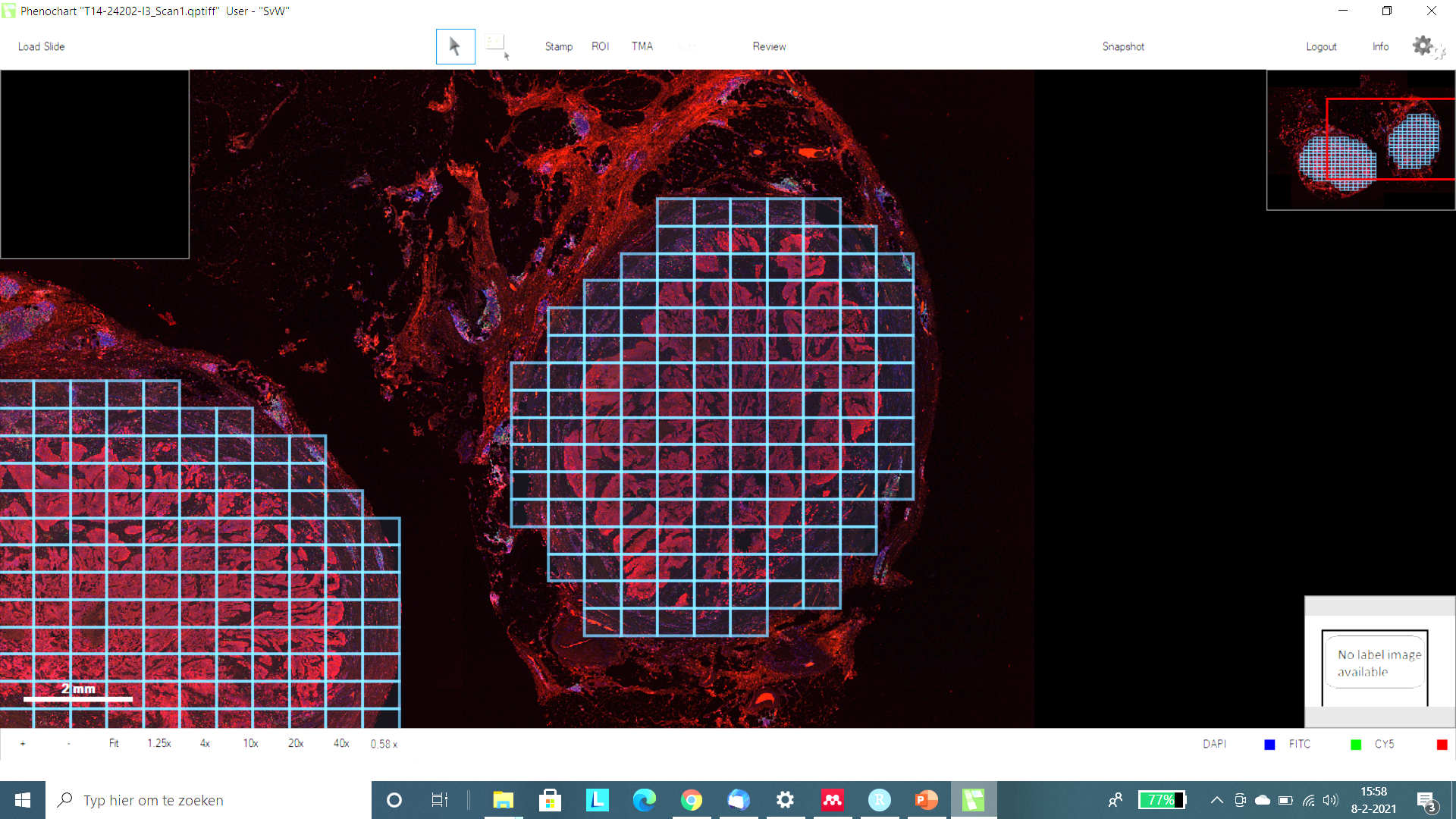
**

**Supplementary figure 1.** Regions of interest. Using the PerkinElmer Phenochart software, tumor regions plus one surrounding region of stroma (669 × 500 µm) were selected for imaging at 20X magnification.

**
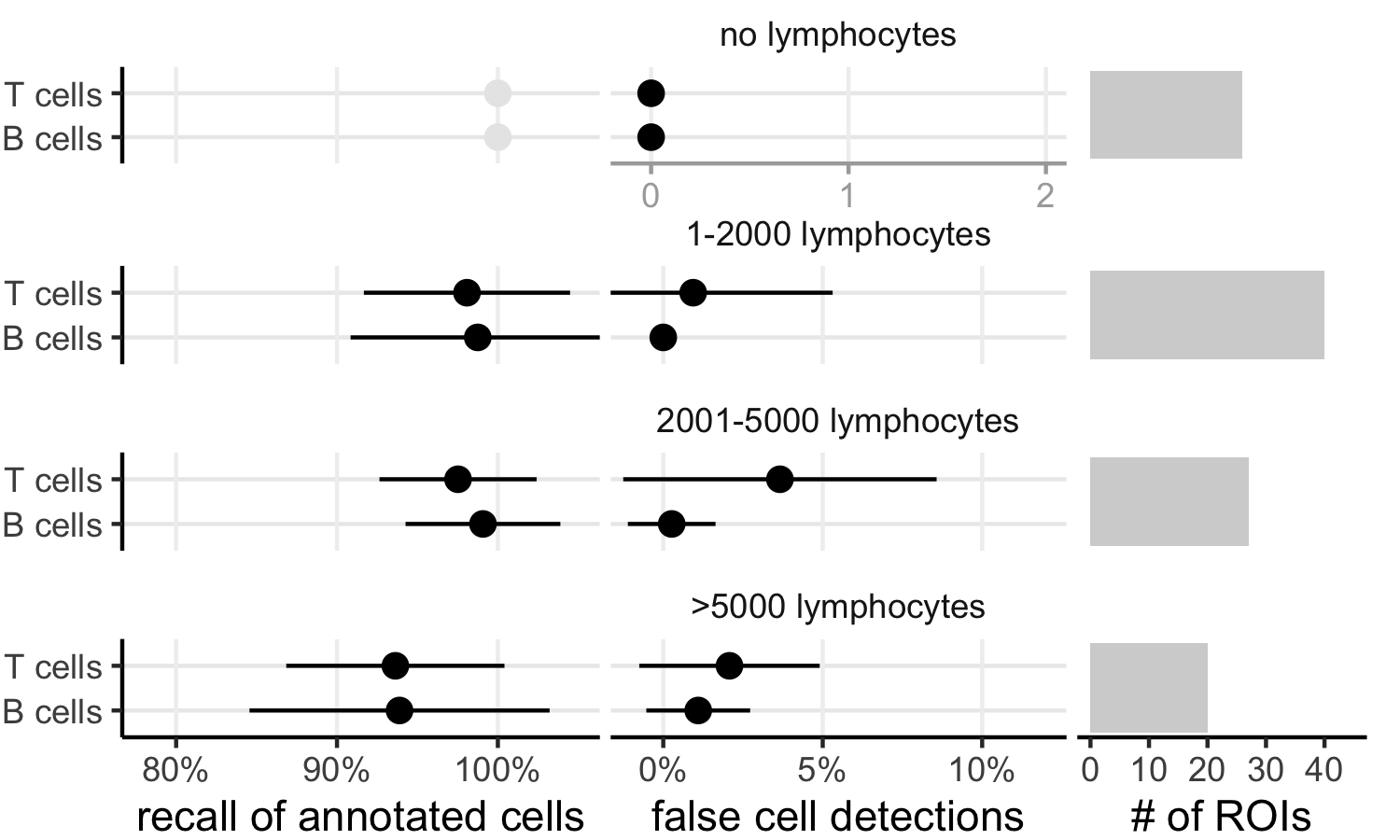
**

**Supplementary figure 2.** Lymphocyte detection accuracy**.** To verify the accuracy of our algorithm in this dataset, we fully annotated 113 regions of 65 µm^2^ (ROI) for T and B cells. Cell detections were considered valid if there was an annotation within 3.5 µm distance. We measure the percentage of annotated cells being detected, and the number of false cell detections (fraction of ROI cell density if lymphocytes were present; absolute count per mm^2^ for empty ROIs). In high density areas, the accuracy of the detected cell positions was slightly lower, though the number of cells detected per ROI remained extremely accurate (T cell ICC: 1.0, B cell ICC: 1.0). Datapoints indicate mean values, errorbars indicate one standard deviation.


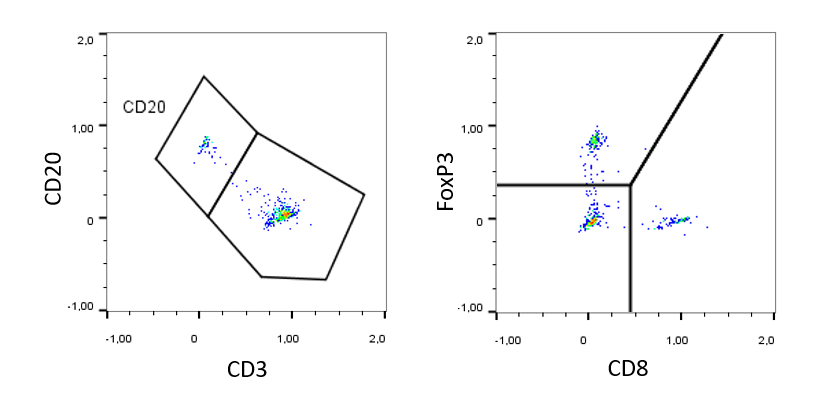


**Supplementary figure 3.** Cell gating in FlowJo based on the neural network’s phenotype prediction. Immune cells were first gated into CD3^+^ and CD20^+^ cells (left panel). CD3^+^ T cells were then subdivided into CD8^+^ T cells, FoxP3^+^ T cells and other cells (right panel).


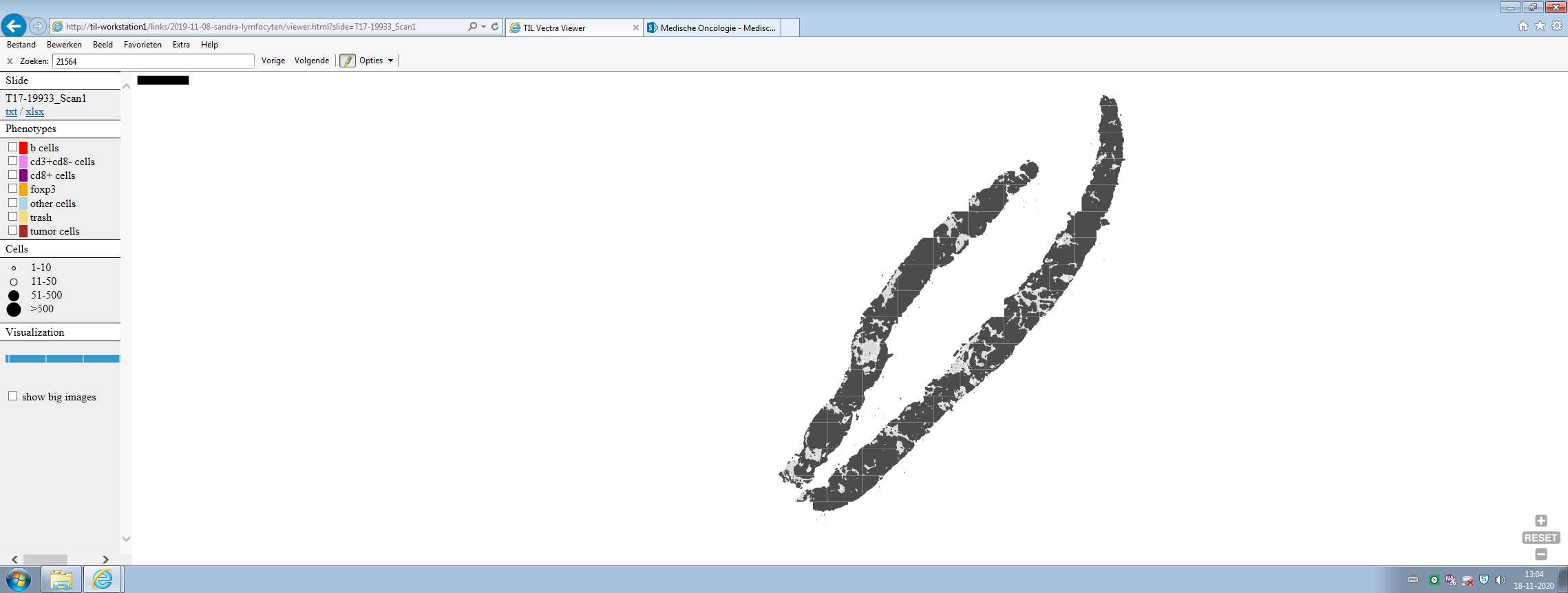


**Supplementary figure 4.** Selection of regions. Dark grey areas depict tissue that is segmented as tumor tissue by inForm, light grey areas depict tissue that is segmented as stroma.


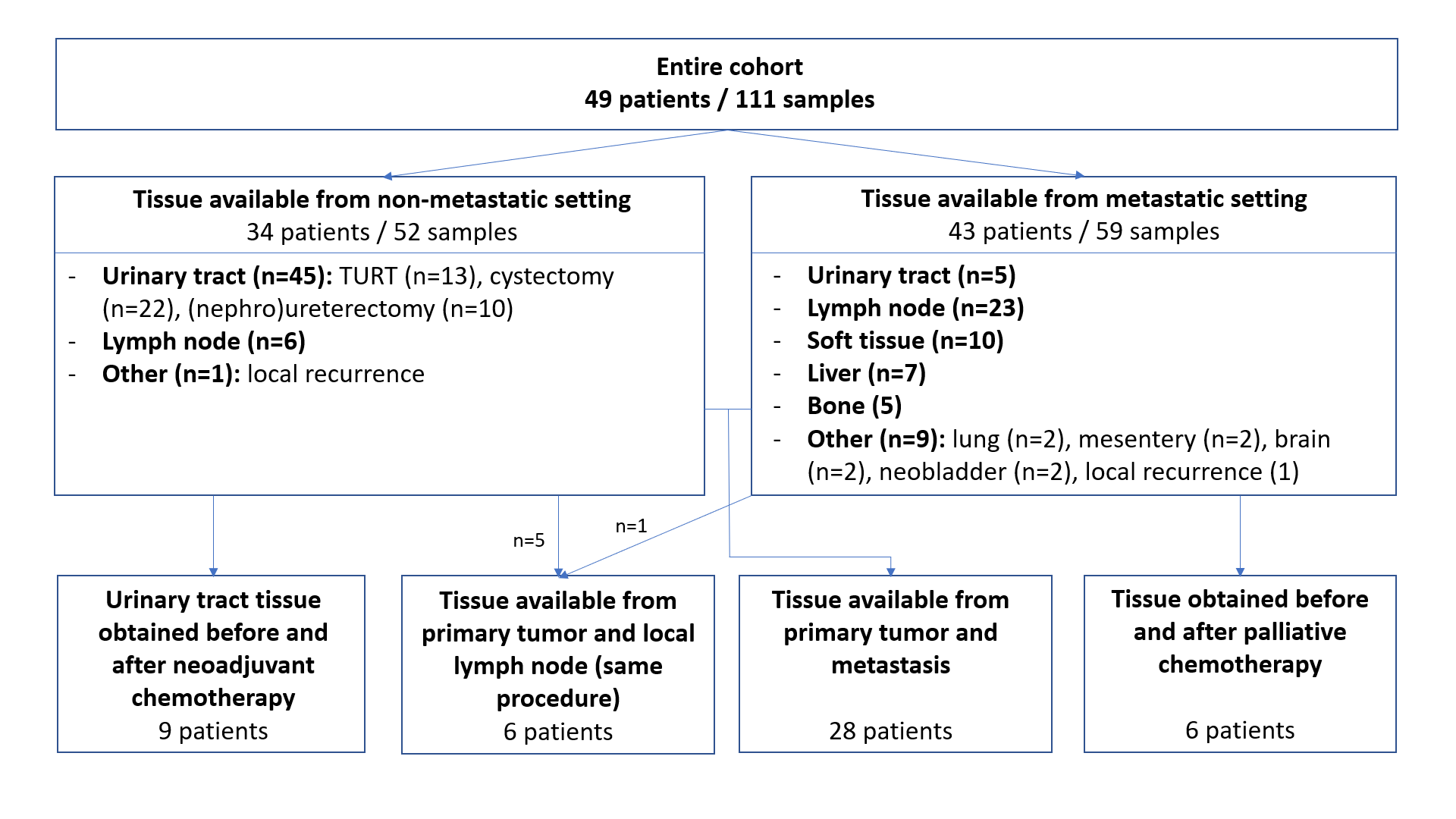


**Supplementary figure 5.** Overview of the cohort. In total, 52 samples of 34 patients were available from the non-metastatic setting: 6 patients had both tissue from a primary tumor and a local lymph node available; 9 patients had primary tumor tissue available obtained before and after neoadjuvant chemotherapy; 2 patients had tissue available from two subsequent primary tumors; 1 patient had tissue from the primary tumor and a local recurrence available. In six patients, tissue from the primary tumor and a local lymph node metastases was resected during the same procedure. One of these patients already had a solitary pulmonary metastasis at the time of surgery for which he received radiotherapy postoperatively. One patients underwent a lymph node biopsy prior to neoadjuvant chemotherapy and a (nephro)uretectomy after chemotherapy. This patients was not included in the neoadjuvant subcohort or the primary tumor/lymph node comparison. In total, 59 samples of 43 patients were available from the metastatic setting. Ten and three patients had two and three samples available, respectively.

**
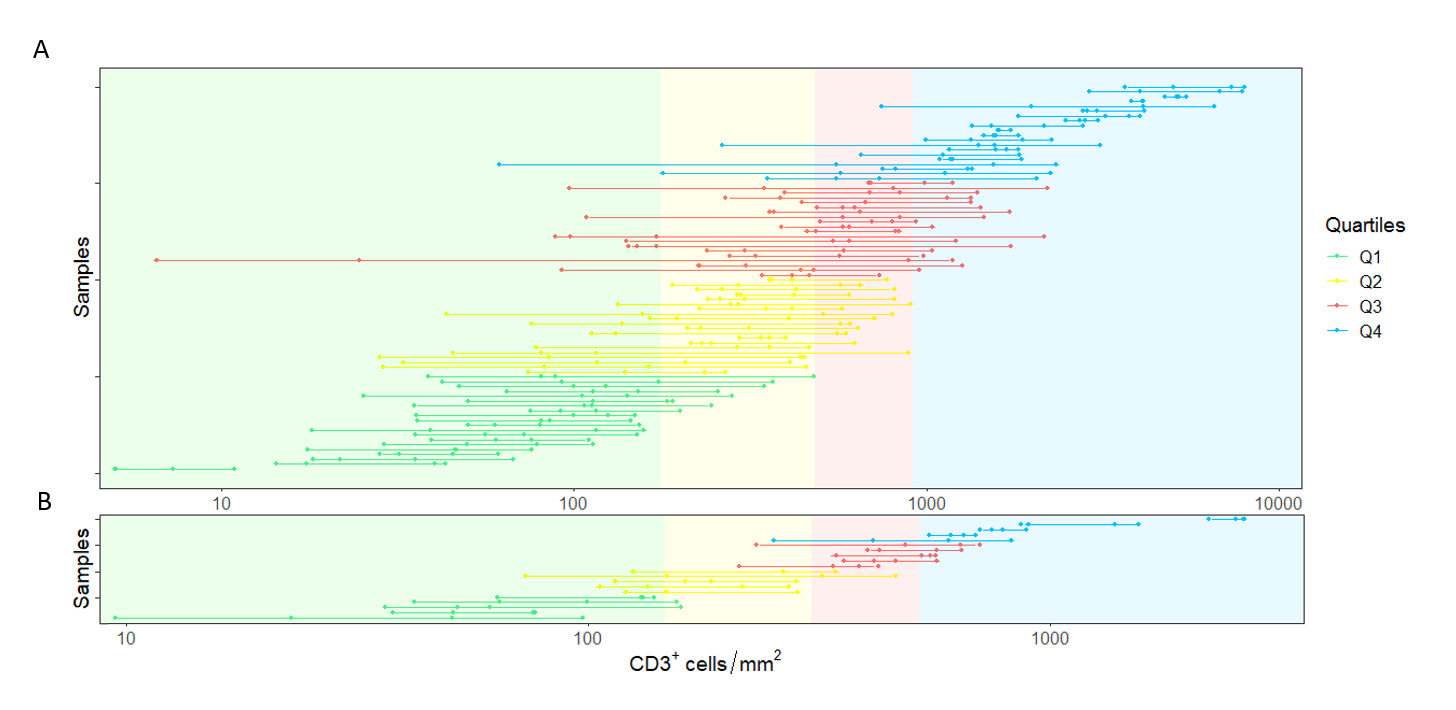
**

**Supplementary figure 6.** CD3^+^ T cell densities in the selected 0.28 mm^2^ **(A)** and 3.30 mm^2^ **(B)** regions. In each sample, four tumor regions were selected. The dots show the cell densities of the regions. The four regions of one sample are interconnected by a line. In some regions, no CD3^+^ cells were present. To enable visualization of cell densities on a log scale, the CD3^+^ densities of these patients was replaced by 5 cells/mm^2^ (~lowest value in A).


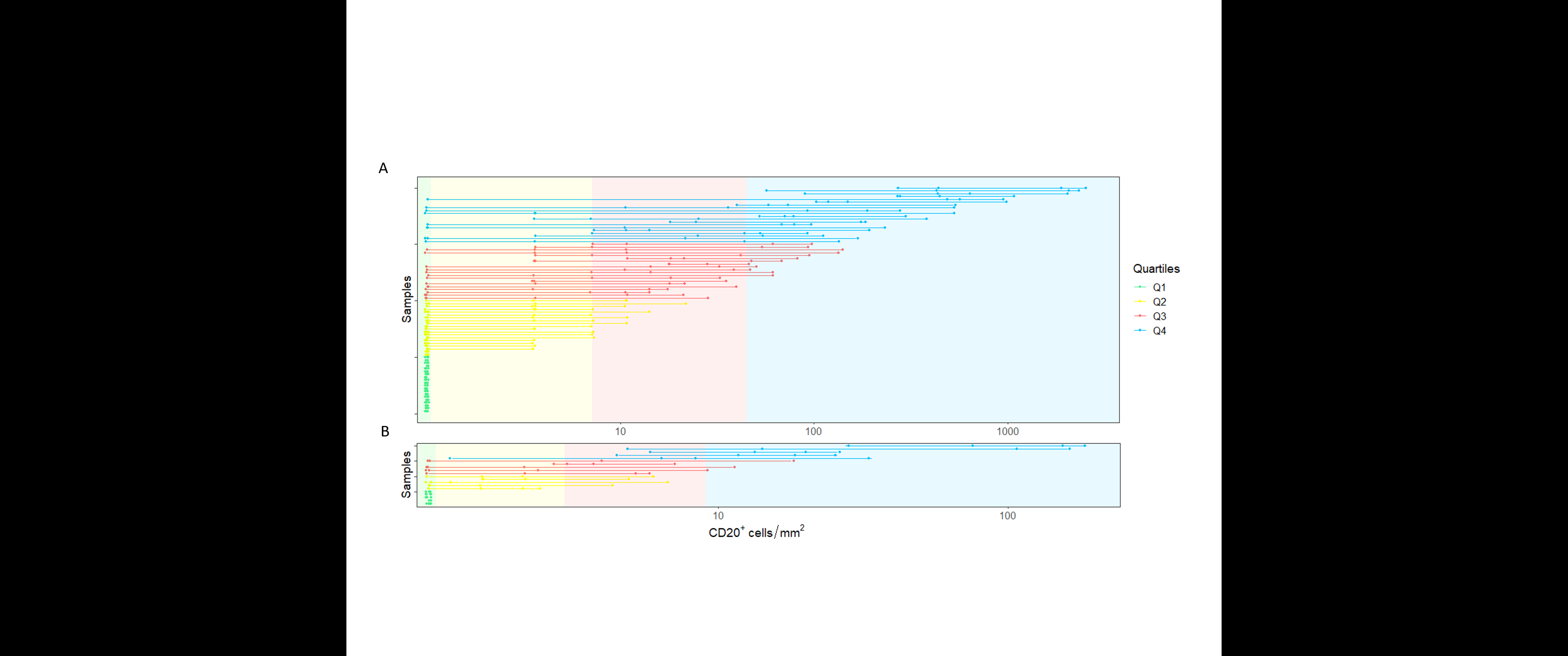


**Supplementary figure 7.** CD20^+^ cell densities in the selected 0.28 mm^2^ **(A)** and 3.30 mm^2^ **(B)** regions. In each sample, four tumor regions were selected. The dots show the cell densities of the regions. The four regions of one sample are interconnected by a line. In some regions, no CD20^+^ cells were present. To enable visualization of cell densities on a log scale, the CD20^+^ densities of these patients was replaced by 1 cells/mm^2^ (~lowest value in A and B). The mean CD20^+^ density of all green-colored samples in A was 0 cells/mm^2^.


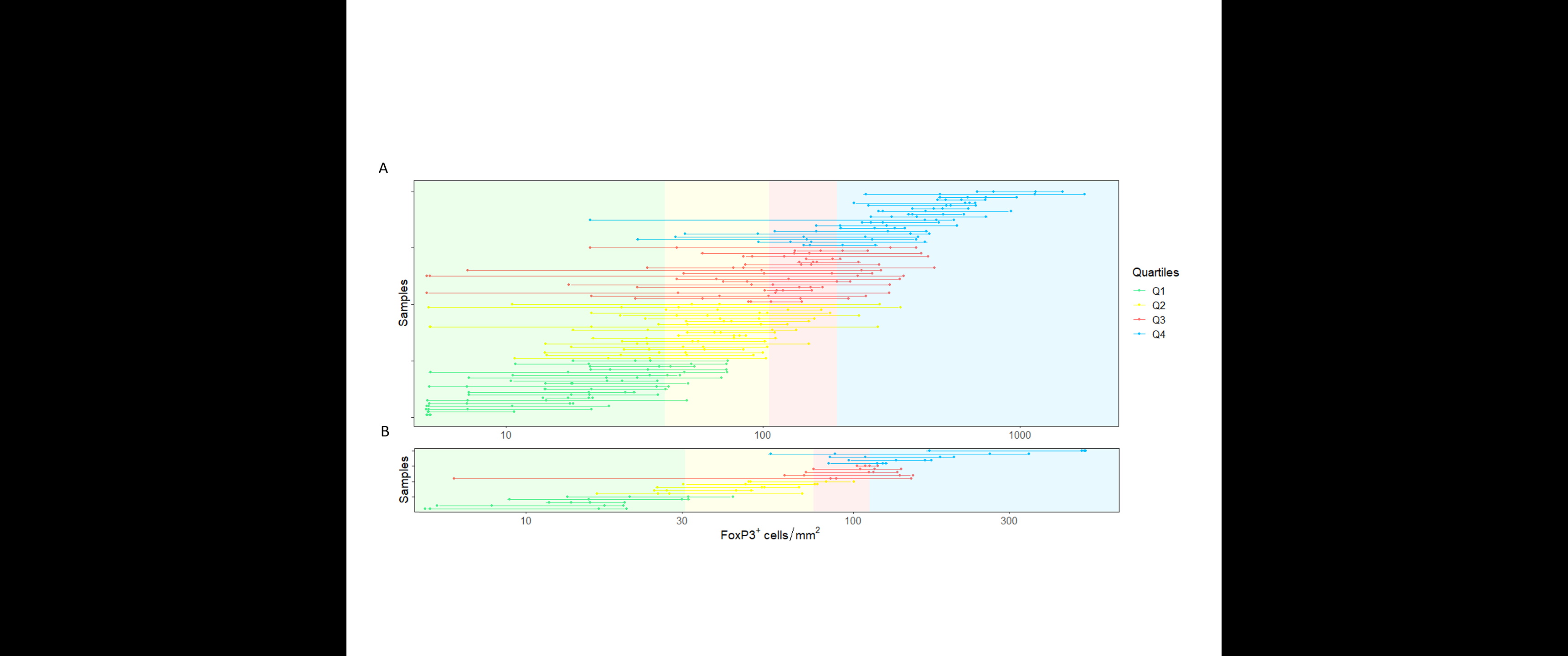


**Supplementary figure 8.** FoxP3^+^ Tcell densities in the selected 0.28 mm^2^ **(A)** and 3.30 mm^2^ **(B)** regions. In each sample, four tumor regions were selected. The dots show the cell densities of the regions. The four regions of one sample are interconnected by a line. In some regions, no FoxP3^+^ cells were present. To enable visualization of cell densities on a log scale, the FoxP3^+^ densities of these patients was replaced by 5 cells/mm^2^ (~lowest value in A and B).


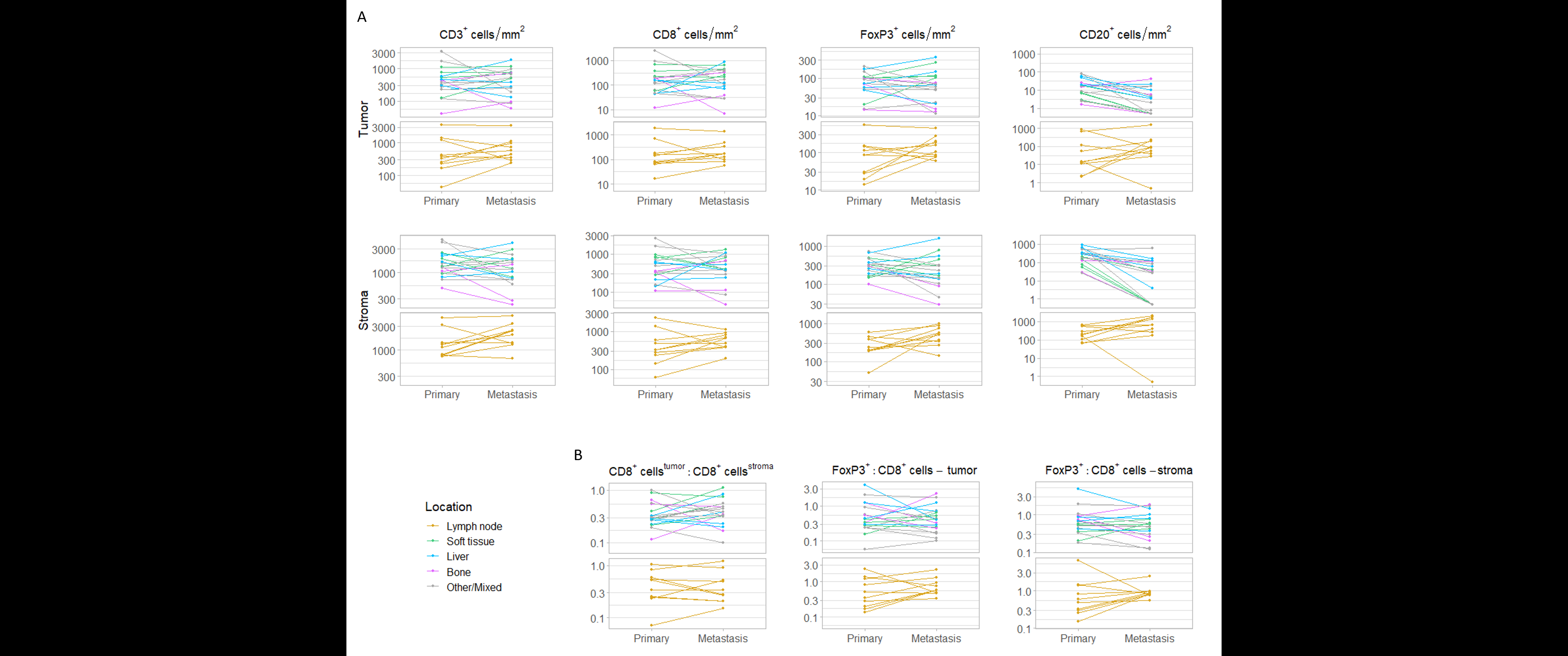


**Supplementary figure 9**. Differences between untreated primary tumor and metachronous distant metastasis. **(A)** CD3^+^, CD8^+^, FoxP3^+^ and CD20^+^ cell density in the tumoral (upper panel) and stromal compartment (lower panel). **(B)** Ratio between cell subsets depicted in A. Each graph is split based on the location where the metastatic tissue was derived from (above = non-lymphoid tissues; below = lymph node metastases). Although cell densities in lymph node metastases seem to be slightly higher compared to the primary tumor, overall, no significant difference in cell density was observed for any of the subsets (Wilcoxon signed-rank test, p<0.05)


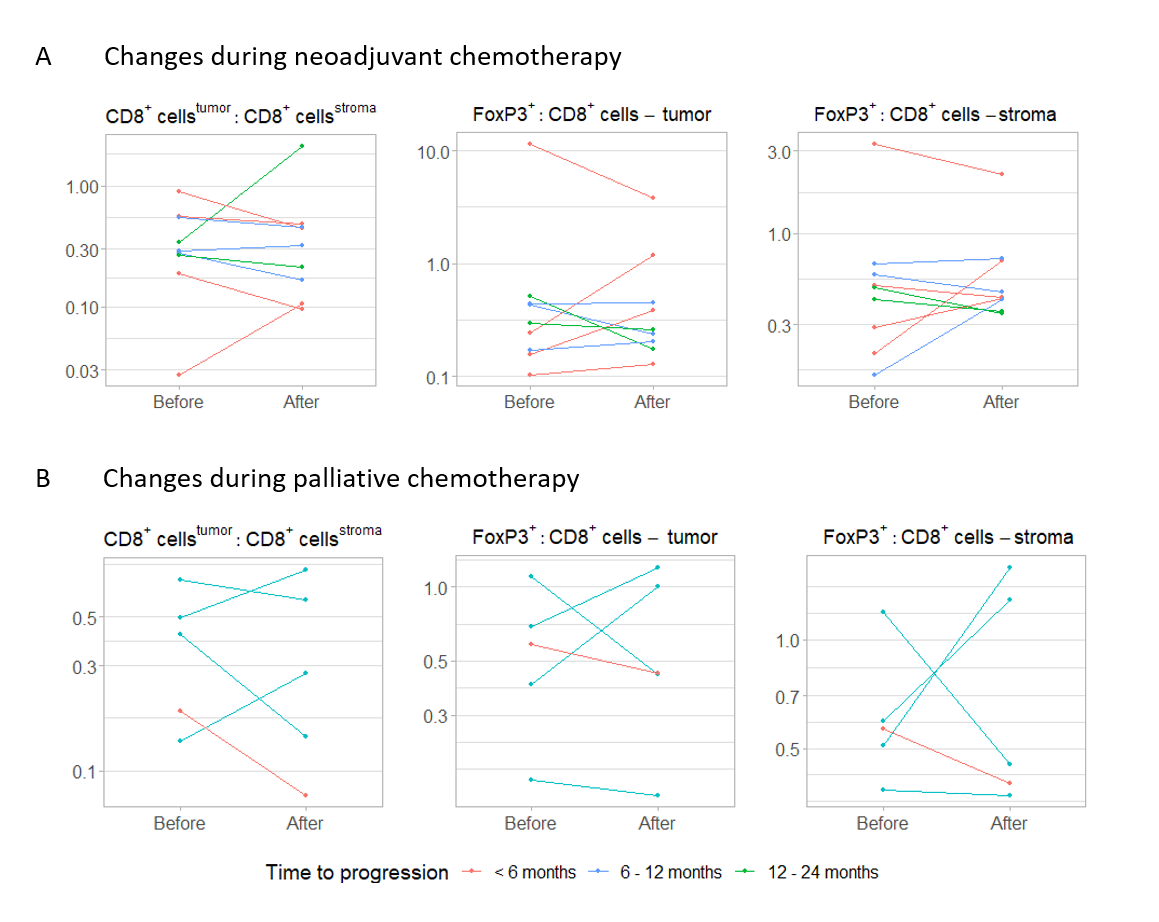


**Supplementary figure 10.** Changes in cell ratios during chemotherapy. **(A)** Changes during neoadjuvant chemotherapy. Samples were obtained by transurethral resection (before) and cystectomy (after). **(B)** Changes during palliative platinum-based chemotherapy. Paired samples were obtained from the same tissue site.
